# Integrative genomic and functional analysis of RAB3B in lung adenocarcinoma: associations with m6A modification and ceRNA networks

**DOI:** 10.3389/fphar.2026.1849560

**Published:** 2026-06-29

**Authors:** Xu-Sheng Liu, Xi Chen, Ning Wang, Zhi-Ming Cheng, Yi-Chi Xie, Ting Jiang, Zhi-Jun Pei

**Affiliations:** Department of Nuclear Medicine, The Second Affiliated Hospital of Soochow University, Suzhou, Jiangsu, China

**Keywords:** ceRNA, lung adenocarcinoma, m6A, prognostic markers, RAB3B

## Abstract

**Background:**

Lung adenocarcinoma (LUAD) is characterized by high mortality and recurrence. This study investigated the clinical significance and biological role of *RAB3B* as a potential biomarker in LUAD.

**Methods:**

We analyzed The Cancer Genome Atlas and Gene Expression Omnibus datasets for *RAB3B* differential expression, prognosis, and correlation with clinical features. Immunohistochemistry validated RAB3B protein levels in tissues. qRT-PCR was employed to detect *RAB3B* mRNA expression in lung cancer cell lines and a normal bronchial epithelial cell line. Following shRNA-mediated knockdown of *RAB3B*, cell proliferation, colony formation, and migration were assessed *via* CCK-8, colony formation, and wound healing assays. Furthermore, we examined *RAB3B*’s correlation with m6A regulatory genes and constructed a *RAB3B*-centric ceRNA network.

**Results:**

*RAB3B* was significantly overexpressed in LUAD tissues. High *RAB3B* levels correlated with pathological stages and male gender. *In vitro*, qRT-PCR confirmed that *RAB3B* mRNA expression was markedly upregulated in lung cancer cell lines. Furthermore, RAB3B protein levels were validated by IHC to be significantly higher in malignant tissues than in normal controls. Functional assays revealed that silencing *RAB3B* significantly inhibited the proliferation, colony formation, and migratory capacity of cancer cells. Additionally, *RAB3B* expression was positively correlated with the m6A “reader” gene *IGF2BP3*. A ceRNA network highlighted hsa-miR-15b-5p as a key downregulated miRNA targeting *RAB3B*, with several upregulated lncRNAs potentially sponging hsa-miR-15b-5p to enhance *RAB3B* expression.

**Conclusion:**

*RAB3B* is a prognostic biomarker and promotes LUAD progression by enhancing cell proliferation and motility. This process likely involves *IGF2BP3* and a miR-15b-5p-related ceRNA mechanism, highlighting *RAB3B* as a promising biomarker or candidate for further therapeutic study.

## 1 Introduction

As a primary histological variant of non-small cell lung cancer, lung adenocarcinoma (LUAD) has increased in incidence in recent years (Mantilla and Moreira, 2024). International data from the World Health Organization further confirms that lung malignancies continue to be the primary factor in cancer-associated mortality on a global scale (Sung et al., 2021; Thai et al., 2021; Pachimatla et al., 2024). Although current treatments for LUAD, including surgery, radiotherapy, and chemotherapy, are available, their effectiveness is often limited by poor therapeutic outcomes and high recurrence rates (Seguin et al., 2022; Wu et al., 2023; Zhang et al., 2023). Consequently, identifying novel biomarkers and therapeutic targets is of critical importance (Guo et al., 2022; TANG et al., 2023; Zheng et al., 2023; Liu et al., 2026).

Recent studies have linked aberrant *RAB3B* expression to tumor development and progression in several cancers. These findings strongly imply that *RAB3B* may have prognostic relevance in cancer (Klengel et al., 1997; Luo et al., 2021; Tsunedomi et al., 2022; Liu et al., 2024a; Xu et al., 2024). Existing data suggest that increased levels of *RAB3B* are closely linked to poor survival rates in LUAD cases, marking it as a valuable candidate for prognostic assessment (Yao et al., 2024). This association underscores why this specific protein is considered a significant biological indicator for predicting disease progression in clinical settings. Additionally, the development and malignancy of LUAD are significantly influenced by m6A methylation and competitive endogenous RNA (ceRNA) networks. These regulatory mechanisms contribute to the development and progression of LUAD. m6A modification regulates tumor-related gene expression by influencing RNA stability, splicing, and translation (Yu et al., 2023; 2024). Previous research has highlighted the significant role of the ceRNA network in cancer biology by regulating miRNA activity, which in turn affects gene expression and contributes to cancer development (Liu et al., 2024b; 2025c; Si et al., 2025). However, no studies have yet explored the potential link between *RAB3B*, m6A methylation and ceRNA in LUAD. Exploring the roles of *RAB3B* within these regulatory networks could clarify the molecular origins of LUAD. Such insights are essential for identifying innovative targets that facilitate more effective clinical diagnosis and therapeutic interventions for patients.

In the current research framework, we used bioinformatics and statistical analyses to examine public transcriptomic datasets. This encompassed differential expression profiling and prognostic survival analysis, leveraging high-throughput data retrieved from The Cancer Genome Atlas (TCGA) and the Gene Expression Omnibus (GEO) repositories. Subsequently, to provide empirical corroboration for these preliminary computational predictions, immunohistochemistry (IHC) assays were executed to assess the protein abundance of *RAB3B* within clinical tissue specimens. Parallel to this, quantitative real-time PCR (qRT-PCR) and a series of *in vitro* functional experiments were conducted to verify gene expression levels and elucidate the specific biological roles of *RAB3B* using lung adenocarcinoma (LUAD) cell lines. Through this integrative strategy, which synergizes the analysis of large-scale public datasets with wet-lab experimental validation, we aim to comprehensively explore the underlying biological functions and the potential clinical significance of *RAB3B* in the context of LUAD. This integrated approach can reveal correlations between gene expression patterns and clinical phenotypes, providing critical insights for future research and clinical applications. Additionally, we will examine the relationship between *RAB3B*, m6A regulation, and ceRNA. A thorough investigation of *RAB3B*’s functions and its impact on cell proliferation and migration holds promise for advancing early diagnosis and personalized treatment strategies for LUAD.

## 2 Materials and methods

### 2.1 Dataset acquisition

Data for this study were retrieved from the TCGA LUAD dataset (https://portal.gdc.cancer.gov/), yielding a cohort of 539 malignant and 59 healthy tissues (Tomczak et al., 2015; Liu et al., 2018). From the GEO platform (www.ncbi.nlm.nih.gov/geo), we also gathered the GSE31210 series, which features 226 cancerous and 20 control specimens (Barrett et al., 2012). Furthermore, survival and follow-up data for an additional 442 tumor subjects were extracted from the GSE72094 dataset within the same GEO archive.

### 2.2 Differential expression analysis of *RAB3B* mRNA in LUAD

The identification of differentially expressed genes (DEGs) within the TCGA-LUAD dataset was executed utilizing the DESeq2 package (version 1.36.0) (Love et al., 2014) in the R software (version 4.5.1; R Foundation for Statistical Computing, Vienna, Austria; https://www.r-project.org/). In parallel, the limma package (version 3.66.0) (Ritchie et al., 2015) was selected to process the microarray data derived from the GSE31210 dataset. To ensure the reliability of the screening process, we defined statistically significant genes using a threshold of an adjusted P-value (P.adj) < 0.05 and a Log fold change (LogFC) > 0.58. Furthermore, the Wilcoxon rank-sum test was employed to compare the mRNA levels of *RAB3B* between cancerous tissues and healthy control groups in both study cohorts. Ultimately, all relevant statistical findings were graphically visualized using the ggplot2 package (version 4.0.1) (Wickham, 2016).

### 2.3 Prognostic and diagnostic value analysis of *RAB3B* in LUAD

To assess the prognostic role of *RAB3B*, we conducted survival regression analyses on three independent cohorts: TCGA LUAD, GSE31210, and GSE72094. To assess the influence of specific variables on overall survival (OS), we constructed univariate Cox proportional hazards models. This statistical evaluation was performed using the R ‘survival' package (version 3.8.3) (Therneau and Grambsch, 2000), focusing on OS as the primary clinical outcome for the study cohort. Based on the median *RAB3B* mRNA levels, the study population was categorized into low- and high-expression cohorts. We then employed log-rank tests to compare survival outcomes between these groups. A Hazard Ratio (HR) > 1 alongside a P < 0.05 defined statistical significance, identifying genes linked to elevated mortality risk. ROC curve analysis for the TCGA LUAD and GSE31210 datasets was performed using the pROC package (version 1.18.0) (Robin et al., 2011), with results visualized *via* ggplot2.

### 2.4 Correlation analysis between *RAB3B* mRNA expression and clinical pathological characteristics

To determine the relationship between *RAB3B* mRNA levels and key clinicopathological variables, we performed correlation analyses within the TCGA LUAD dataset. Utilizing the ‘stats (version 4.2.1)' and ‘car (version 3.1–0)' packages in R, we specifically evaluated patient demographics such as age and gender, alongside diagnostic indicators like tumor stage. These associations were subsequently visualized through the ‘ggplot2' framework.

### 2.5 Sample processing and RAB3B protein detection *via* IHC

Formalin-fixed paraffin-embedded (FFPE) human LUAD tissue samples were obtained from ZuoLi Biotechnology Co., Ltd (Shanghai, China). This cohort consisted of 48 malignant tissues and 48 paired peritumoral normal tissues. The immunohistochemical (IHC) staining procedures were conducted following a standard protocol previously described in the literature, ensuring experimental consistency and reproducibility (Liu et al., 2025a; Qu et al., 2025). Briefly, 4-μm thick paraffin-embedded tissue sections were deparaffinized in xylene (three times for 10 min each) and rehydrated through a series of graded ethanol (100%, 95%, 85%, and 75% for 5 min each). Antigen retrieval was performed using citrate buffer (pH 6.0) in a pressure cooker. After blocking endogenous peroxidase with 3% H_2_O_2_ and nonspecific binding with 5% goat serum, tissue slices underwent incubation with a Rabbit Anti-RAB3B/C Polyclonal antibody (1:50, 15774-1-AP, Proteintech) overnight at 4 °C. For visual detection, the Dako REAL EnVision Detection System, Peroxidase/DAB+ (K5007, Dako, Glostrup, Denmark) was applied, with hematoxylin utilized as a counterstain to clarify nuclear structures. Images were captured *via* light microscopy and quantitatively analyzed using Fiji/ImageJ software (version 1.53t, National Institutes of Health, Bethesda, MD, United States of America) to evaluate the RAB3B protein levels.

### 2.6 Cell lines and culture conditions

For our experimental models, we selected three specific human cell lines: the normal bronchial epithelial BEAS-2B (iCell-h023, iCell, China), together with lung cancer cell lines HCC827 (iCell-h068, iCell, China) and NCI-H460 (iCell-h160, iCell, China). While BEAS-2B cells were sustained in DMEM (12,634,010, Gibco, United States of America), the HCC827 and NCI-H460 lines were cultivated in RPMI 1640 medium (C11875500BT, Gibco, United States of America). All growth media were supplemented with 10% fetal bovine serum (FBS; 10099141C, Gibco, United States of America) and 1% penicillin-streptomycin (P/S; 15,140–122, Gibco, United States of America). Standardized culture conditions were maintained in a 3111 CO_2_ incubator (Thermo Scientific, United States of America) at 37 °C with 5% CO_2_ and relative humidity. For routine maintenance and subculturing, cells at 80%–90% confluence were detached using 0.25% Trypsin-EDTA (C25200056, Gibco, United States of America).

### 2.7 Cell transfection

Short hairpin RNAs (shRNAs) targeting *RAB3B* (shRAB3B#1, #2, and #3) and a negative control shRNA (shNC) were synthesized. The target sequences for *RAB3B* were as follows: shRAB3B#1: 5′-CTG​GGA​CAA​TGC​ACA​AGT​TAT-3'; shRAB3B#2: 5′-GCT​TCA​GTG​ACA​GAT​GGT​AAA-3'; and shRAB3B#3: 5′-TGG​GCT​TCA​TTC​TGA​TGT​ATG-3'. Transfections were performed to establish experimental groups for subsequent functional characterization.

### 2.8 Quantitative real-time PCR (qRT-PCR)

Total RNA was harvested from the cultured cells with the RNAiso Easy reagent (TCH020, Takara, Japan). Subsequent to this, the PrimeScript™ RT Reagent Kit (RR047A, Takara, Japan) was utilized to transcribe the RNA into cDNA. The expression levels were quantified through qRT-PCR using TB Green® Premix Ex Taq™ (RR420A, Takara, Japan) on the Gentier 96 Real-Time PCR platform (Tianlong, China). The amplification conditions were as follows: initial denaturation at 95 °C for 30 s, followed by 40 cycles of 95 °C for 5 s and 60 °C for 30 s. For this analysis, the following primer sequences were employed: *RAB3B* (forward: 5′-CTG​TCC​AAG​ACT​GGG​CTA​CTC-3'; reverse: 5′-AAA​CCC​AAG​CTG​CTC​TGC​AA-3′) and β-actin (forward: 5′-GAT​GAG​ATT​GGC​ATG​GCT​TT-3'; reverse: 5′-GTC​ACC​TTC​ACC​GTT​CCA​GT-3′). The 2^−ΔΔCt^ technique was applied to determine relative mRNA abundance, using β-actin as the endogenous reference for normalization.

### 2.9 Cell proliferation assay

Cell growth capacity was assessed through the Cell Counting Kit-8 (CCK-8; KGA317; Nanjing KeyGen Biotech Co., Ltd., China) method. Initially, cells were distributed into 96-well microplates at a uniform concentration of 5 × 10^3^ per well. Following incubation for designated intervals (0, 24, 48, and 72 h), 10 µL of the CCK-8 solution was added to the respective wells and maintained at 37 °C for another 4 h. The resulting cell viability was then evaluated by measuring the absorbance at 450 nm, utilizing a Multiskan FC microplate reader (Thermo Fisher Scientific, United States of America) to record the data. Statistical analysis was conducted using two-way repeated measures ANOVA followed by Bonferroni *post hoc* correction for multiple comparisons.

### 2.10 Colony formation assay

After the transfection process, cells were harvested and replated into 12-well plates at a fixed density of 800 units per well. Upon the formation of observable cell clusters, the growth medium was discarded, and the plates were cleaned with PBS (C10010500BT, Gibco, United States of America). To stabilize and visualize the colonies, we applied 4% paraformaldehyde (P0099, Beyotime, China) for a 20-min fixation period, subsequently staining the samples with 0.1% crystal violet (C0121, Beyotime, China) for an equal duration. Digital records of the stained colonies were then obtained and processed using ImageJ software to determine the colony counts.

### 2.11 Wound healing assay

For the migration assay, cells were plated into 6-well plates at a concentration of 5 × 10^5^ cells/well. After the cell layer attained complete confluence, a simulated wound was generated by scratching the monolayer with a sterile 200 µL pipette tip. To eliminate detached cellular debris, the wells were rinsed with PBS before being replenished with serum-deprived medium. Migration progress was monitored *via* an inverted microscope (CKX53, Olympus, Japan), with photographs taken at specific intervals of 0, 24, and 48 h.

### 2.12 Correlation and functional enrichment analysis of co-expressed genes with *RAB3B* in LUAD

To assess the molecular consequences of *RAB3B*, patients within the TCGA LUAD database were stratified into high and low cohorts based on their respective mRNA abundance. This classification allowed for a comparative analysis between the two expression groups to further elucidate the gene’s functional role in lung adenocarcinoma. Using these defined cohorts, we performed a differential expression analysis *via* the R-based DESeq2 software package. Furthermore, the association between *RAB3B* and various protein-coding genes within the same dataset was evaluated by calculating Pearson correlation coefficients. Heatmaps and lollipop diagrams were generated using ggplot2 to display the top 10 upregulated and downregulated genes. Genes with LogFC >1 and Cor >0, or LogFC < −1 and Cor <0, and P. adj <0.05 were identified as co-expressed genes. To elucidate the biological significance of the identified genes, functional enrichment analysis was executed through the clusterProfiler R package (Yu et al., 2012). The underlying pathways and biological processes derived from this analysis were subsequently illustrated using the ggplot2 visualization library to ensure clear data representation.

### 2.13 Correlation analysis between *RAB3B* mRNA expression and m6A regulation in LUAD

We utilized data from the TCGA LUAD, GSE31210, and GSE30219 cohorts to evaluate the association between *RAB3B* and m6A-related regulatory genes *via* Pearson correlation coefficients. To delve deeper into these molecular interactions, the study population was divided into two distinct clusters using the median *RAB3B* mRNA level as the threshold. This grouping enabled a comparative analysis of how m6A regulators varied between these two distinct expression profiles. UpSet diagrams were used to identify target genes meeting these criteria. The lists of m6A regulatory-related genes were referenced from previous studies (Li et al., 2018; 2019; Liu X.-S. et al., 2023).

### 2.14 Systematic prediction and screening of *RAB3B*-associated ceRNA regulatory components

To comprehensively explore the potential regulatory mechanisms of *RAB3B* in LUAD, we first conducted a prediction analysis of candidate microRNAs (miRNAs) were predicted using multiple online databases. Specifically, *RAB3B* was used as the core target gene in the platforms microT-CDS (https://dianalab.e-ce.uth.gr/html/dianauniverse/index.php?r=microT_CDS), miRWalk (http://mirwalk.umm.uni-heidelberg.de/), miRNet (https://www.mirnet.ca/miRNet/home.xhtml), miRDB (https://mirdb.org/mirdb/index.html), miRmap (https://mirmap.ezlab.org/app), and TarBase-v9.0 (https://dianalab.e-ce.uth.gr/tarbasev9), ENCORI (https://rnasysu.com/encori/index.php) to identify potential miRNAs with binding affinity. This multi-database approach was used to increase screening stringency. As each database employs distinct predictive algorithms and includes experimentally validated datasets, their integrative usage significantly enhanced the precision and biological credibility of miRNA identification. Subsequently, a stringent differential expression analysis (|LogFC| > 1 and P < 0.05) was conducted using TCGA-LUAD transcriptomic data to eliminate miRNAs with inconsistent expression or lacking statistical significance, thereby establishing a high-specificity candidate miRNA set.

Following miRNA screening, we proceeded to identify potential upstream long non-coding RNAs (lncRNAs) that may regulate *RAB3B* by competitively binding the selected miRNAs. For this step, we relied on the ENCORI and miRNet platforms, using miRNA binding site predictions and expression co-variation analysis to select lncRNA candidates. The differential expression criteria were consistent with those used in miRNA screening, ensuring that the identified lncRNAs exhibited clear expression trends and regulatory potential, consistent with a competitive endogenous RNA (ceRNA) model involving *RAB3B* in LUAD.

Based on the competitive endogenous RNA (ceRNA) theory, we established a molecular interaction map focused on *RAB3B*. This framework assumes that lncRNAs and mRNAs can modulate each other’s activity by competing for a limited pool of microRNAs through mutual miRNA response elements (MREs). To validate the molecular foundation of key interactions within this predicted network, we employed the miRanda algorithm (version 3.3a) (Enright et al., 2003) to analyze sequence pairing between all candidate miRNAs and their associated mRNAs and lncRNAs. By identifying potential binding sites, we supplemented the ceRNA model with structural evidence, reinforcing the credibility of the predicted network at both theoretical and molecular levels.

### 2.15 Statistical analysis

All computational analyses were conducted using the R programming language, where the DESeq2 and limma packages were utilized for the identification of differentially expressed genes in the TCGA and GEO datasets. To compare *RAB3B* levels between tumor and healthy specimens, we applied the Wilcoxon rank-sum test. Survival outcomes were evaluated by stratifying the patient population into two cohorts using the median *RAB3B* expression as the cutoff, followed by the application of Kaplan-Meier curves and log-rank tests. Furthermore, univariate Cox regression models were constructed to determine the Hazard Ratios (HR) and associated P values. Visualization of the data was partially facilitated by the Xiantao Online Tool (https://www.xiantaozi.com/), and a P-value <0.05 was consistently employed as the threshold for determining statistical significance.

## 3 Results

### 3.1 Diagnostic and prognostic significance of high *RAB3B* mRNA expression in LUAD

**FIGURE 1:**
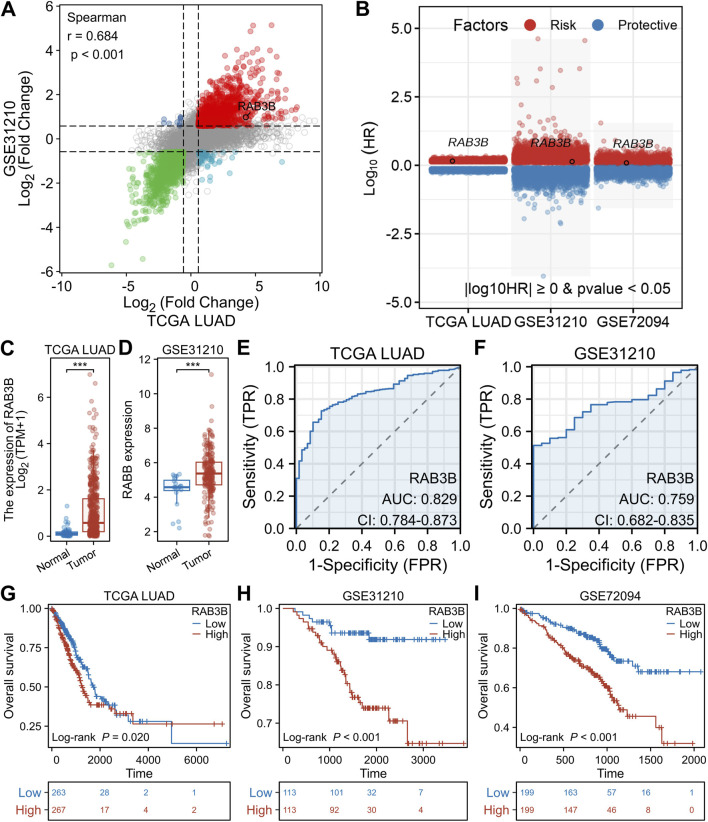
Diagnostic and prognostic significance of high expression of *RAB3B* mRNA in LUAD. **(A)** A cross-dataset differential expression scatter plot depicting common transcriptomic alterations within the TCGA LUAD and GSE31210 series, showing upregulation of *RAB3B* in malignant *versus* healthy tissues. A concordant expression pattern of RAB3B across the two datasets was further confirmed by Spearman correlation analysis. **(B)** A forest-style grouped scatter plot highlighting the prognostic relevance of *RAB3B* across TCGA LUAD, GSE31210, and GSE72094 cohorts, as evaluated by univariate Cox modeling. **(C,D)** Boxplots illustrating the marked elevation of *RAB3B* transcript levels in cancerous specimens relative to control groups for both TCGA LUAD and GSE31210 databases. **(E,F)** ROC analysis providing evidence for the diagnostic utility of *RAB3B* in distinguishing LUAD cases. **(G–I)** Kaplan-Meier plots revealing a significant association between increased *RAB3B* mRNA levels and diminished overall survival (OS) among individuals diagnosed with LUAD.

A cross-dataset differential expression scatter plot was constructed to evaluate the consistency of differential gene expression patterns between the TCGA-LUAD and GSE31210 datasets. This integrated mapping approach allowed us to categorize and compare the patterns of gene expression changes across these two independent study populations simultaneously. This analysis identified that *RAB3B* exhibited consistently elevated expression levels in malignant tissues relative to healthy controls in both independent datasets (TCGA: LogFC = 4.254, P. adj <0.05; GSE31210: LogFC = 0.977, P. adj <0.05) (Figure 1A). Spearman correlation analysis demonstrated a consistent expression trend of *RAB3B* across the TCGA LUAD and GSE31210 datasets (Cor = 0.684, P < 0.001) (Figure 1A). Quantitative comparisons between groups confirmed that *RAB3B* was significantly upregulated in cancerous tissues *versus* normal controls across both study cohorts (Figures 1C,D). Additionally, the prognostic relevance of *RAB3B* was confirmed *via* univariate Cox proportional hazards modeling across three independent cohorts. Specifically, elevated *RAB3B* mRNA levels were significantly associated with adverse clinical outcomes in the TCGA LUAD, GSE31210, and GSE72094 datasets (HR = 1.407, log10(HR) = 0.148, P < 0.05; HR = 1.344, log10(HR) = 0.129, P < 0.05; HR = 1.245, log10(HR) = 0.095, P < 0.05) (Figure 1B). By stratifying patients based on median expression, Kaplan-Meier analysis further illustrated that the high-expression arm had shorter overall survival (OS) compared to those with lower expression levels (Figures 1G–I,P < 0.05). Furthermore, ROC curves showed diagnostic performance of *RAB3B*, yielding area under the curve (AUC) values of 0.829 (95% CI: 0.784–0.873) for the TCGA cohort and 0.759 (95% CI: 0.682–0.835) for the GSE31210 series (Figures 1E,F).

### 3.2 Association between *RAB3B* and clinicopathological features in LUAD patients

**FIGURE 2:**
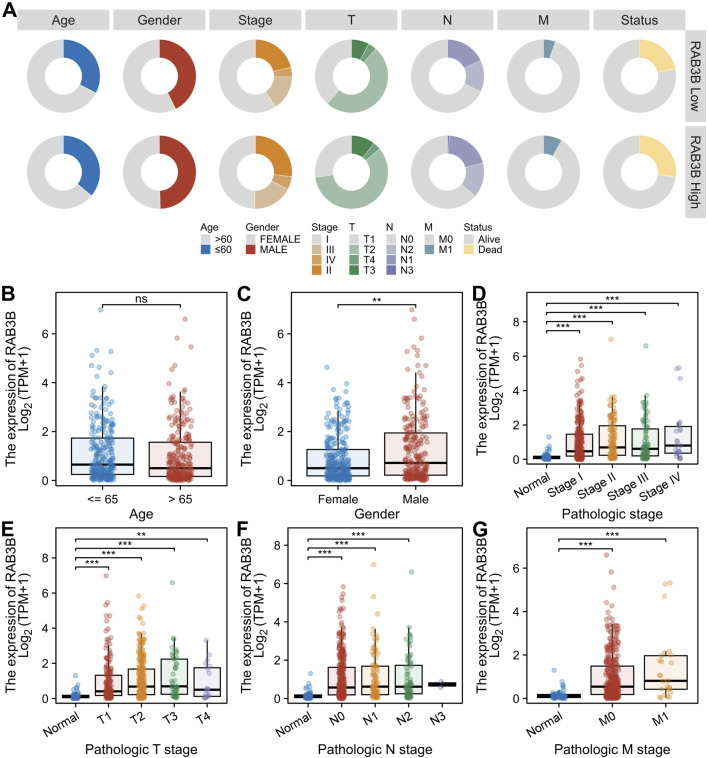
Clinical correlation and pathological analysis of *RAB3B* mRNA expression in TCGA LUAD dataset. **(A)** Doughnut charts illustrating the relationship between various clinicopathological factors and *RAB3B* mRNA abundance within the TCGA LUAD cohort. **(B)** A boxplot indicating that *RAB3B* transcript levels are not significantly influenced by patient age, as evidenced by a P-value >0.05 **(C)** Comparative analysis of *RAB3B* mRNA expression by gender, showing significantly increased levels in male subjects (P < 0.05). **(D–G)** Set of boxplots illustrating that *RAB3B* mRNA expression is consistently and significantly upregulated across various pathological classifications, including pathological stage and T, N, and M stages, when compared to baseline normal samples (P < 0.05).

A heatmap was generated to illustrate the distribution of clinical features across different *RAB3B* mRNA expression strata within the TCGA LUAD cohort (Figure 2A). Statistical assessments indicated that while *RAB3B* mRNA expression remained independent of patient age (Figure 2B, P > 0.05), a distinct correlation with gender was observed, with male patients showing higher levels than females (Figure 2C, P < 0.05). Furthermore, consistent elevation of *RAB3B* was documented across various clinico-pathological parameters, including pathological stage and T, N, and M categories, relative to healthy tissues (Figures 2D–G, P < 0.05).

### 3.3 RAB3B is significantly upregulated in LUAD tissues and exhibits diagnostic potential

**FIGURE 3:**
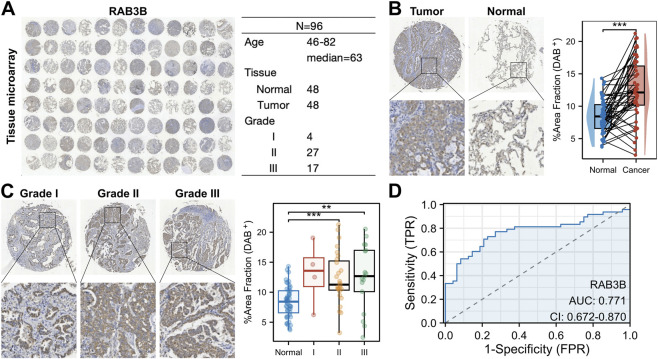
Immunohistochemical analysis and diagnostic evaluation of RAB3B protein expression in LUAD. **(A)** Overview of LUAD tissue microarray IHC staining and associated clinical data. **(B)** Representative IHC images and statistical comparison showing significantly elevated RAB3B protein expression in LUAD tissues *versus* normal lung tissues (P < 0.05). **(C)** Stratified analysis indicating higher RAB3B protein expression in both Grade II and Grade III tumors compared to normal tissues (P < 0.05). **(D)** ROC curve demonstrating the diagnostic value of RAB3B protein expression in LUAD (AUC = 0.771, 95% CI: 0.672–0.870).

As illustrated in Figure 3A, an overview of the LUAD tissue microarray IHC staining and corresponding clinical data of patients was presented. Representative IHC images of both normal lung tissues and LUAD specimens are shown in Figure 3B. Statistical evaluations indicated a significant increase in RAB3B protein abundance within malignant specimens relative to the corresponding peritumoral lung tissues (Figure 3B, P < 0.05). When further categorized by histological differentiation, both Grade II and Grade III LUAD tissues exhibited markedly higher RAB3B protein levels than control groups (Figure 3C, P < 0.05), implying that this gene’s upregulation is intrinsically linked to disease advancement. Additionally, ROC curve assessments reinforced the diagnostic utility of RAB3B, yielding an area under the curve (AUC) of 0.771 (95% CI: 0.672–0.870), which reflects a robust capacity for distinguishing between diseased and healthy states (Figure 3D).

### 3.4 Impact of *RAB3B* knockdown on the proliferation and migration of lung cancer cells

**FIGURE 4:**
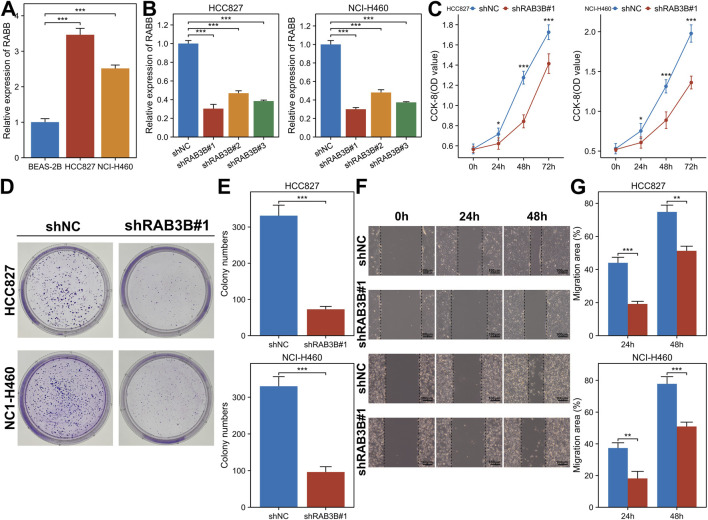
Regulatory effects of *RAB3B* depletion on lung cancer cell proliferation and motility. **(A)** Analysis of *RAB3B* mRNA abundance across the BEAS-2B, HCC827, and NCI-H460 cell populations. **(B)** Validation of the interference efficiency in HCC827 and NCI-H460 cells following transfection with three independent shRAB3B sequences (#1, #2, and #3) using qRT-PCR. **(C)** CCK-8 profiles tracking the growth kinetics of HCC827 and NCI-H460 cells in the shRAB3B#1 and shNC cohorts over a 72-h period. Data were analyzed using two-way repeated measures ANOVA followed by Bonferroni *post hoc* correction for multiple comparisons. **(D,E)** Typical micrographs and corresponding statistical assessment of colony-forming units in HCC827 and NCI-H460 cells after *RAB3B* silencing. **(F,G)** Representative scratch assay images and subsequent migration rate quantification showing the impact of *RAB3B* knockdown on cellular movement.

To investigate the clinical relevance of *RAB3B* in lung cancer, its expression levels were measured in various cell lines. The qRT-PCR results indicated that *RAB3B* mRNA expression was significantly upregulated in HCC827 and NCI-H460 lung cancer cell lines compared with the normal bronchial epithelial cell line BEAS-2B (P < 0.05) (Figure 4A). To silence *RAB3B*, three specific shRNAs were transfected into HCC827 and NCI-H460 cells. Results from qRT-PCR demonstrated that the mRNA abundance of *RAB3B* was markedly diminished in the shRAB3B#1, shRAB3B#2, and shRAB3B#3 cohorts compared to the shNC control group (P < 0.05, Figure 4B). Based on the knockdown efficiency, shRAB3B#1 was selected for the following functional assays.

To characterize the regulatory function of *RAB3B* in cellular growth, we performed CCK-8 and colony formation experiments. The CCK-8 data indicated that the viability of HCC827 and NCI-H460 cells in the shRAB3B#1 cohort was markedly suppressed at 24, 48, and 72 h in comparison to the shNC control (P < 0.05, Figure 4C). In line with these findings, the colony formation assay showed a significant decrease in the number of cell clusters within the shRAB3B#1 group relative to the shNC group (P < 0.05, Figures 4D,E), underscoring the critical role of *RAB3B* in maintaining the proliferative potential of lung cancer cells.

Furthermore, we conducted a wound healing assay to evaluate how *RAB3B* affects the migratory capacity of HCC827 and NCI-H460 cells. Typical microscopy images revealed that the recovery of the denuded area was significantly slower in the shRAB3B#1 group than in the shNC group (Figure 4F). Statistical evaluation further verified that the migration velocity of HCC827 and NCI-H460 cells was substantially reduced following the depletion of *RAB3B* (P < 0.05, Figure 4G), which implies that *RAB3B* is a key driver of lung cancer cell motility *in vitro*.

### 3.5 Co-expressed genes and functional enrichment analysis of *RAB3B* in LUAD

**FIGURE 5:**
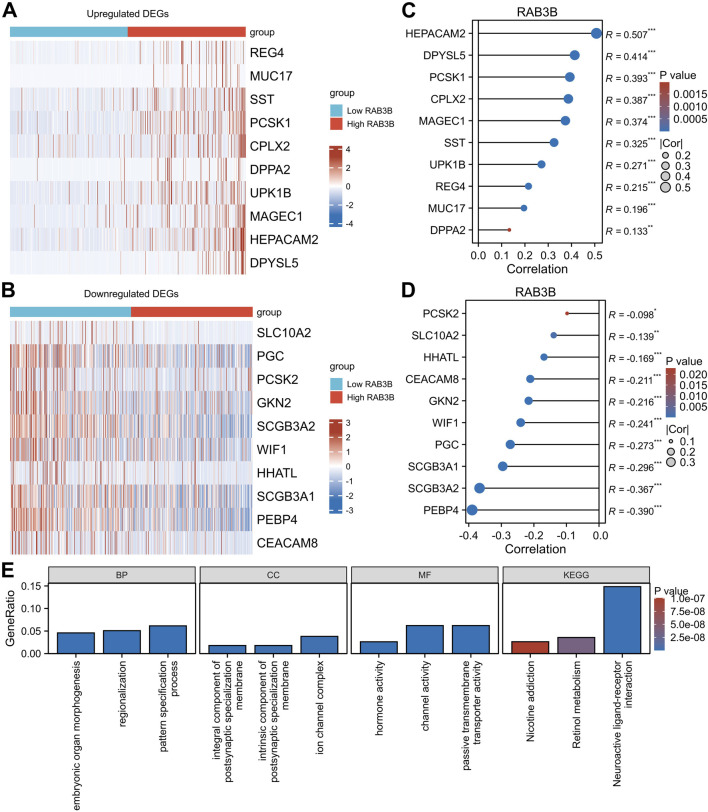
Differentially expressed genes and functional enrichment analysis associated with *RAB3B* in LUAD. **(A,B)** Heatmaps illustrating the expression profiles of the 10 most significantly increased and decreased genes between the *RAB3B*-high and *RAB3B*-low subsets within the TCGA LUAD cohort. **(C,D)** Lollipop plots demonstrating the Pearson correlation coefficients between *RAB3B* levels and the top 10 genes from the up- and downregulated categories. **(E)** Functional clustering through GO and KEGG enrichment, emphasizing the association of co-expressed genes with pattern specification, membrane constituents of postsynaptic specialization, and ion channel activity, alongside the Neuroactive ligand-receptor interaction signaling pathway.

To characterize the molecular mechanisms associated with *RAB3B* in LUAD, we conducted a comparative transcriptomic analysis between high-expression and low-expression cohorts within the TCGA LUAD database. This identification of differentially expressed genes (DEGs) served as the foundation for further elucidating the potential biological roles of this target gene. Heatmaps displayed the top 10 upregulated and downregulated genes under the criteria |LogFC|>1 and P. adj<0.05 (Figures 5A,B). Pearson correlation analysis evaluated the relationships between *RAB3B* and these genes, with bar plots illustrating their correlations (Figures 5C,D). A total of 1,364 co-expressed genes were identified based on the criteria LogFC>1 and Cor>0, or LogFC<−1 and Cor<0, with P. adj<0.05. GO and KEGG enrichment analyses of these genes revealed significant involvement in biological processes such as pattern specification, integral component of postsynaptic specialization membrane, channel activity, and the neuroactive ligand-receptor interaction pathway (Figure 5E).

### 3.6 Correlation analysis between *RAB3B* mRNA expression and m6A regulation

**FIGURE 6:**
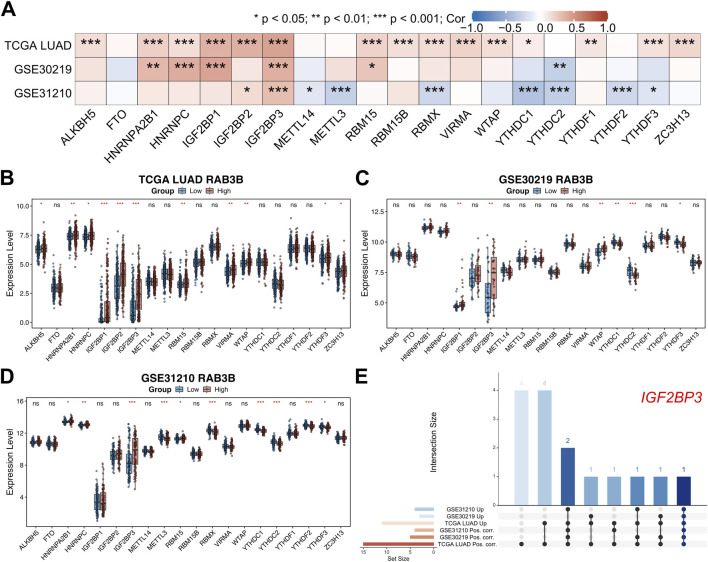
Interrelationship between *RAB3B* abundance and m6A-regulatory genes in LUAD within various study series. **(A)** Heatmaps illustrating the correlation between *RAB3B* mRNA levels and 20 m6A-associated regulators across the TCGA LUAD, GSE31210, and GSE30219 archives. **(B–D)** Boxplots demonstrating the marked elevation of *IGF2BP3* transcripts in the *RAB3B*-high cohort relative to the *RAB3B*-low subset in the TCGA LUAD, GSE31210, and GSE30219 databases. **(E)** UpSet plots identifying *IGF2BP3* as a consistently significant factor exhibiting a robust overlap across different analytical platforms.

To explore the potential link between *RAB3B* abundance and m6A-mediated methylation in LUAD, we performed an integrated assessment across the TCGA LUAD, GSE31210, and GSE30219 archives. Pearson correlation coefficients indicated that *RAB3B* mRNA expression was positively correlated with 15 m6A regulators within the TCGA LUAD cohort (Figure 6A, P < 0.05). Notably, a consistent positive correlation was identified between *RAB3B* and *IGF2BP3* across both the GSE31210 and GSE30219 datasets (Figure 6A, P < 0.05). Further transcriptomic comparisons demonstrated that *IGF2BP3* levels were markedly elevated in the *RAB3B*-high subset relative to the *RAB3B*-low group across all three independent series (Figures 6B–D, P < 0.05). Additionally, the UpSet plot analysis prioritized *IGF2BP3* as a key molecule exhibiting a significant statistical relationship with *RAB3B* (Figure 6E).

### 3.7 Construction of *RAB3B*-centric ceRNA network and screening of regulatory miRNAs

**FIGURE 7:**
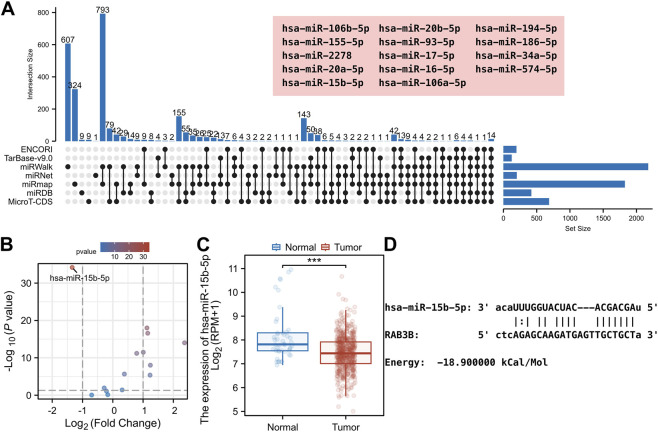
Identification of *RAB3B*-targeting miRNAs and validation of hsa-miR-15b-5p as a key regulator in LUAD. **(A)** UpSet plot showing the overlap of miRNAs predicted to target *RAB3B* across seven databases (microT-CDS, miRWalk, miRNet, miRDB, miRmap, TarBase v9.0, and ENCORI), revealing 14 shared candidates. **(B)** Comparative transcriptomic profiling of the 14 candidate miRNAs within the TCGA LUAD database, which pinpointed six significantly increased and one specifically decreased (hsa-miR-15b-5p) miRNA in malignant specimens. **(C)** Expression validation of hsa-miR-15b-5p showing significant downregulation in LUAD samples, suggesting a negative regulatory relationship with *RAB3B*. **(D)** Predicted binding site between hsa-miR-15b-5p and *RAB3B* identified using the miRanda tool, supporting their direct interaction at the sequence level.

To examine potential regulatory mechanisms of *RAB3B* in LUAD, we constructed a ceRNA interaction map centered on *RAB3B*. This integrated network was designed to delineate the intricate crosstalk between non-coding RNAs and the *RAB3B* transcript, thereby elucidating its post-transcriptional control. A systematic miRNA screening was conducted using multiple widely recognized databases, including microT-CDS, miRWalk, miRNet, miRDB, miRmap, TarBase v9.0, and ENCORI. These platforms predicted 685, 420, 1826, 201, 2,178, 126, and 197 miRNAs potentially interacting with *RAB3B*, respectively. UpSet plot analysis (Figure 7A) revealed that 14 miRNAs were consistently identified across all seven databases, representing a high-confidence candidate set.

Subsequently, the transcriptomic profiles of these 14 candidate miRNAs were evaluated for differential expression within the TCGA LUAD database (Figure 7B). This comparative assessment aimed to validate the specific dysregulation patterns of these small non-coding RNAs between malignant and healthy tissues. The analysis showed that six miRNAs were significantly upregulated in tumor tissues: hsa-miR-20a-5p (LogFC = 1.01, P = 3.290e-12), hsa-miR-20b-5p (LogFC = 1.23, P = 4.303e-06), hsa-miR-93–5p (LogFC = 1.13, P = 1.011e-18), hsa-miR-17–5p (LogFC = 1.15, P = 2.641e-17), hsa-miR-106a-5p (LogFC = 1.24, P = 9.747e-09), and hsa-miR-194–5p (LogFC = 2.37, P = 9.654e-15). Only hsa-miR-15b-5p (LogFC = −1.34, P = 6.417e-35) was found to be downregulated. According to the ceRNA hypothesis, upregulated mRNAs are typically associated with reduced expression of their targeting miRNAs in tumors. Based on this principle, hsa-miR-15b-5p was selected as the most promising candidate for targeting *RAB3B*, which was confirmed to be downregulated in tumor samples (Figure 7C).

To further investigate their interaction, we utilized the miRanda tool for sequence pairing analysis and identified a potential binding site between hsa-miR-15b-5p and *RAB3B* (Figure 7D), providing molecular-level evidence to support their regulatory relationship.

### 3.8 Candidate ceRNA lncRNAs mediated by hsa-miR-15b-5p identified *via* dual-database screening and expression profiling

**FIGURE 8:**
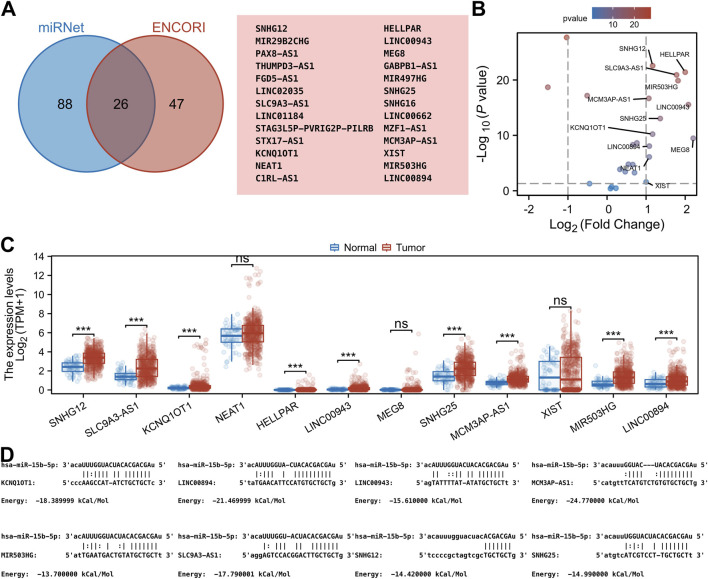
Identification and characterization of hsa-miR-15b-5p-associated lncRNAs potentially regulating *RAB3B*. **(A)** An intersection analysis visualized *via* a Venn diagram, identifying 26 mutual lncRNAs predicted as hsa-miR-15b-5p targets by both the ENCORI and miRNet algorithms. **(B)** A volcano plot depicting the transcriptomic distribution of these candidate lncRNAs in the TCGA LUAD database, which prioritized 12 significantly increased molecules for further investigation. **(C)** Box plots displaying the expression levels of nine upregulated lncRNAs significantly overexpressed in tumor *versus* normal tissues (P < 0.05). **(D)** MiRanda-based sequence pairing analysis revealing putative binding sites between hsa-miR-15b-5p and the eight candidate lncRNAs, supporting their involvement in ceRNA regulation. HELLPAR was excluded due to incomplete transcript annotation.

To identify lncRNAs that may competitively bind hsa-miR-15b-5p and participate in *RAB3B* regulation, we conducted target prediction using ENCORI and miRNet. These platforms yielded 73 and 114 lncRNAs, respectively. An intersection analysis (Figure 8A) revealed 26 overlapping candidates, suggesting a subset of highly credible lncRNAs co-predicted across tools.

Differential expression analysis using the TCGA LUAD dataset revealed that 12 of these candidates were significantly upregulated in tumors, including: SNHG12 (LogFC = 1.17, P = 2.682e-23), SLC9A3-AS1 (LogFC = 1.78, P = 1.207e-21), KCNQ1OT1 (LogFC = 1.17, P = 6.130e-11), NEAT1 (LogFC = 1.08, P = 7.950e-07), HELLPAR (LogFC = 2.00, P = 3.923e-22), LINC00943 (LogFC = 2.08, P = 2.825e-16), MEG8 (LogFC = 2.21, P = 3.423e-10), SNHG25 (LogFC = 1.36, P = 8.969e-14), MCM3AP-AS1 (LogFC = 1.07, P = 2.085e-17), XIST (LogFC = 1.00, P = 2.652e-02), MIR503HG (LogFC = 1.82, P = 1.314e-20), and LINC00894 (LogFC = 1.08, P = 8.877e-09). Meanwhile, LINC02035 (LogFC = −1.03, P = 2.130e-28) and MIR497HG (LogFC = −1.51, P = 2.023e-19) were significantly downregulated (Figure 8B).

According to the ceRNA hypothesis, where lncRNAs and mRNAs typically show co-expression patterns, the two downregulated lncRNAs (LINC02035 and MIR497HG) were excluded from the model. Among the 12 upregulated candidates, secondary validation revealed that only 9 (SNHG12, SLC9A3-AS1, KCNQ1OT1, HELLPAR, LINC00943, SNHG25, MCM3AP-AS1, MIR503HG, and LINC00894) exhibited statistically significant differences between tumor and normal tissues (Figure 8C, P < 0.05).

Among the nine significantly upregulated lncRNAs, HELLPAR could not be analyzed further because a complete transcript sequence was unavailable in public databases. Sequence-pairing analysis using miRanda identified putative binding sites for the remaining eight lncRNAs (Figure 8D).

**FIGURE 9:**
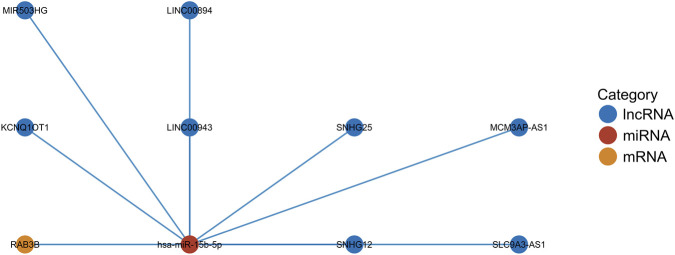
CeRNA network related to RAB3B in LUAD. Diagram illustrating the putative ceRNA mechanism by which SNHG12, SLC9A3-AS1, KCNQ1OT1, LINC00943, SNHG25, MCM3AP-AS1, MIR503HG, and LINC00894 competitively bind hsa-miR-15b-5p, relieving its inhibitory effect on *RAB3B* and enhancing *RAB3B* expression. The model is supported by integrative analyses of expression profiles and miRNA-lncRNA-mRNA sequence complementarity.

Based on the integrative analyses of bioinformatic predictions, expression profiling, and sequence pairing, we propose that SNHG12, SLC9A3-AS1, KCNQ1OT1, LINC00943, SNHG25, MCM3AP-AS1, MIR503HG, and LINC00894 may function as ceRNAs that competitively bind hsa-miR-15b-5p. This interaction potentially reduces the miRNA-mediated repression of *RAB3B*, thereby enhancing *RAB3B* expression (Figure 9). The regulatory associations are in line with the classical ceRNA model and are further supported by expression correlation and sequence complementarity data.

## 4 Discussion

LUAD is a common type of non-small cell lung cancer, and its incidence has been gradually increasing in recent years, causing serious health issues for patients and imposing a significant economic burden on society (Seguin et al., 2022; Pachimatla et al., 2024). Despite the integration of surgical intervention, radiation, and pharmacological therapies into the current therapeutic landscape for LUAD, clinical outcomes frequently remain suboptimal due to high recurrence rates. This persistent challenge underscores a critical imperative to discover novel molecular signatures and actionable targets capable of enhancing the prognostic accuracy and overall survival for individuals diagnosed with this malignancy (Jiang et al., 2024; Liu et al., 2025c; 2025b; Qu et al., 2025).

Our earlier investigations revealed that *RAB3B* is markedly overexpressed across a range of cancer types, and this heightened expression correlates with tumor heterogeneity, the immune microenvironment, and patient prognosis (Liu et al., 2024a). Additionally, studies indicate that *RAB3B* is closely related to the occurrence and development of several cancers, including prostate cancer (Tan et al., 2012), pancreatic cancer (Klengel et al., 1997), liver cancer (Tsunedomi et al., 2022), and glioma (Luo et al., 2021). Limbu’s research points out that the interaction of *RAB3B* with various stem cell-related genes may influence tumor immune cell infiltration and patient survival rates, suggesting that *RAB3B* plays an important role not only in tumor progression but also potentially in the tumor microenvironment (Limbu and McCloskey, 2023). Through the systematic integration of large-scale transcriptomic data from the TCGA and GEO repositories, our research identified a substantial upregulation of *RAB3B* in malignant samples relative to their healthy counterparts. These bioinformatic predictions were further corroborated by IHC staining, which confirmed that RAB3B protein accumulation is significantly more pronounced in LUAD tissues than in non-cancerous specimens. In parallel, qRT-PCR analysis substantiated that *RAB3B* mRNA abundance is markedly increased in various lung cancer cell models compared to normal bronchial epithelial cells. Our functional investigations provided evidence that the genetic silencing of *RAB3B via* shRNA significantly abrogated cellular proliferation, inhibited colony-forming efficiency, and impaired the migratory potential of lung cancer cells *in vitro*. Similar integrated *in vitro* and *in silico* approaches have been successfully employed to characterize the anti-proliferative and anti-metastatic effects of novel therapeutic agents in LUAD cells, emphasizing the importance of targeting cell cycle progression and ROS-mediated pathways (Chandramohan et al., 2025). Furthermore, elevated levels of *RAB3B* expression are closely correlated with unfavorable prognostic outcomes in patients diagnosed with LUAD. These findings collectively imply that *RAB3B* serves as a pivotal driver in the oncogenesis and advancement of LUAD by facilitating malignant cell proliferation and metastatic spread, especially within the frameworks of clinical diagnosis and prognostic stratified evaluation. Such a role is corroborated by recent evidence suggesting that *RAB3B* interacts with the DEAD-box helicase DDX6, which subsequently augments its protein stability and accelerates LUAD progression. This interaction offers further insight into the sophisticated molecular mechanisms by which *RAB3B* modulates tumor biology (Yao et al., 2024).

Notably, our analysis revealed that *RAB3B* mRNA expression was significantly higher in male patients than in females. While the precise reason for this gender-related disparity in LUAD remains unclear, it may be linked to the regulatory effects of sex hormones. Previous studies have indicated that certain Rab family members can be modulated by androgen receptor signaling (Mizushima et al., 2020), which is known to play a role in the progression of various cancers, including lung cancer (Mikkonen et al., 2010). Additionally, gender-specific differences in lifestyle factors or distinct epigenetic profiles between sexes might also contribute to the elevated *RAB3B* levels in males. Further investigations are required to decipher the exact molecular interplay between sex hormones and *RAB3B* regulation. Our functional enrichment analysis reveals that genes co-expressed with *RAB3B* are mainly involved in biological processes such as neuroactive ligand-receptor interaction, further supporting the critical role of *RAB3B* in cell communication and signal transduction. Consistent with our findings, network pharmacology and molecular docking studies have also identified the neuroactive ligand-receptor interaction pathway as a key signaling mechanism in lung cancer regulation (Wu et al., 2021).

Existing literature has demonstrated the important role of m6A methylation in various cancers, particularly in regulating cell metabolism and proliferation (Teng et al., 2021; Yu et al., 2023; 2024). Nevertheless, direct empirical evidence elucidating the intricate interplay between *RAB3B*, m6A epitranscriptomic modifications, and glycolytic flux in LUAD remains sparse. Our current findings reveal a compelling correlation between *RAB3B* mRNA abundance and the landscape of m6A methylation, particularly through its coordinated interaction with the IGF2BP protein family. This observation implies that *RAB3B* might orchestrate the oncogenic progression of LUAD by modulating the expression profiles of key m6A regulators. Further stratified analysis demonstrated a pronounced elevation of *IGF2BP3* in the *RAB3B*-high cohort, which likely underscores the pivotal regulatory role of m6A modifications in maintaining the transcriptional and translational stability of these specific readers. These IGF2BP family members have been shown to promote tumor progression, potentially enhancing the stability and translation efficiency of mRNA to support tumor cell survival and proliferation (Huang et al., 2021; Liu et al., 2022; Fang et al., 2023). Thus, the upregulation of *RAB3B* may influence the expression of IGF2BPs, thereby affecting the m6A methylation process and playing an important role in the pathological process of LUAD.

In the present research, we performed an integrated construction of a *RAB3B*-focused ceRNA network in LUAD by synthesizing multi-platform computational predictions, TCGA-derived transcriptomic profiles, and rigorous sequence complementarity assessments. A key highlight of this regulatory axis is the identification of hsa-miR-15b-5p, which exhibits a distinct downward expression trend in LUAD specimens. This observation aligns with established literature characterizing miR-15b-5p as a potent tumor-suppressive element across diverse malignancies, including colorectal and non-small cell lung carcinomas. In these contexts, miR-15b-5p has been shown to curtail neoplastic growth and trigger programmed cell death by directly repressing oncogenic drivers such as *XIAP* and *PSAT1*(Zhao et al., 2017; Guo et al., 2021). Our study extends its tumor-suppressive role to LUAD, providing the bioinformatic evidence that hsa-miR-15b-5p may directly interact with *RAB3B*, a gene whose oncogenic role is becoming increasingly evident in multiple malignancies. The miRanda-based sequence complementarity further supports a direct regulatory relationship. Notably, the ceRNA network constructed herein highlights several lncRNAs (e.g., SNHG12, KCNQ1OT1, and LINC00943) as potential molecular sponges of hsa-miR-15b-5p. Many of these lncRNAs have independently been implicated in LUAD progression. For instance, SNHG12 has been reported to decrease non-small cell lung cancer (NSCLC) cell sensitivity to cisplatin by repressing miR-525–5p and promoting XIAP expression, thereby enhancing tumor survival under chemotherapeutic stress (Tan et al., 2023). Its oncogenic potential and cytoplasmic localization make it a plausible ceRNA regulator in LUAD contexts. Similarly, KCNQ1OT1 has been shown to facilitate NSCLC cell invasion and migration *via* the miR-496/HMGB1 signaling axis (Wang et al., 2024), suggesting a pro-metastatic role that may intersect with *RAB3B*-mediated pathways. Furthermore, previous functional studies have identified LINC00943 as a pro-oncogenic driver that facilitates LUAD cell proliferation and metastatic dissemination *via* the miR-1252–5p/YWHAH signaling axis (Liu B. et al., 2023). These findings provide a compelling mechanistic precedent for its contribution to malignant progression and underscore the high probability of its integration into a broader ceRNA network alongside *RAB3B*. The concurrent upregulation of these lncRNAs and *RAB3B*, alongside the downregulation of hsa-miR-15b-5p, supports a classical ceRNA mechanism that likely facilitates the derepression of *RAB3B* in LUAD. This model aligns with the ceRNA hypothesis, wherein transcripts such as lncRNAs can competitively bind to shared miRNAs, thereby indirectly modulating mRNA expression through miRNA sequestration. Consequently, our research not only delineates the intricate molecular landscape governing *RAB3B* expression but also postulates a previously unrecognized ceRNA regulatory axis that may fundamentally drive the oncogenic processes in LUAD.

Moreover, our bioinformatics approach, integrating seven miRNA and two lncRNA databases, adds credibility to our network by ensuring a stringent and consensus-driven screening process. This methodological rigor mitigates the high false-positive rate often associated with single-database predictions. Furthermore, as the field evolves from monoomics to spatial multiomics, the integration of cross-platform and cross-modal data analysis frameworks will likely provide even more precise insights into tissue structures and cellular identities in the LUAD microenvironment (Huan et al., 2025). Consequently, subsequent multi-dimensional investigations, encompassing both sophisticated *in vitro* models and *in vivo* preclinical validations, are essential to definitively confirm the physiological significance of this ceRNA regulatory axis. Such research necessitates a deeper exploration into its clinical utility, potentially positioning it as a robust diagnostic signature or a precision therapeutic intervention point for patients with LUAD.

Despite the comprehensive analysis presented, several limitations should be acknowledged. First, while the oncogenic functions of RAB3B were validated in multiple cell lines, the regulatory roles of m6A modifications and the ceRNA network are primarily based on high-throughput bioinformatics and correlation analyses. Due to current resource and funding constraints, direct physical interaction experiments, including dual-luciferase reporter assays and RIP assays, have not yet been performed. Consequently, the sponge mechanism of lncRNAs such as SNHG12 remains a hypothesis that still requires further experimental corroboration. Future studies will focus on performing rescue experiments and molecular interaction assays to definitively validate these post-transcriptional regulatory axes. In addition, the current functional validation was mainly limited to *in vitro* experiments, and *in vivo* studies are still required to further confirm the oncogenic role of RAB3B in LUAD. By integrating the interplay between m6A epitranscriptomic modifications and ceRNA regulatory networks, subsequent research should further elucidate the precise mechanisms by which *RAB3B* dictates the oncogenic evolution of LUAD through the coordinated modulation of these biological processes. This not only helps to reveal the molecular mechanisms of LUAD but may also provide a theoretical basis for developing new therapeutic strategies. Consequently, a comprehensive deconstruction of the regulatory intersection between *RAB3B*, m6A epitranscriptomic modifications, and the ceRNA network may help clarify the regulatory mechanisms in LUAD. Such multidimensional investigations will broaden our conceptual understanding of the tumor’s biological hallmarks, potentially identifying novel vulnerabilities for precision intervention.

## 5 Conclusion

This study systematically characterized the expression pattern, clinical significance, and regulatory mechanisms of *RAB3B* in LUAD. Integrative analyses across multiple datasets, validated by IHC and qRT-PCR, revealed that *RAB3B* is significantly overexpressed in LUAD and associated with pathological features and poor overall survival. Functional assays further demonstrated that *RAB3B* knockdown significantly inhibits cell proliferation, colony formation, and migration, confirming its oncogenic role *in vitro*. Furthermore, *RAB3B* expression is positively correlated with the m6A reader *IGF2BP3* and was associated with a putative ceRNA network involving hsa-miR-15b-5p and specific upregulated lncRNAs, such as SNHG12 and SLC9A3-AS1, which may facilitate *RAB3B* overexpression through miRNA sequestration. Taken together, our findings substantiate the role of *RAB3B* as a clinically significant oncogenic driver and a promising candidate for therapeutic intervention. This study delineates a sophisticated layer of epitranscriptomic and post-transcriptional regulation governing LUAD advancement, thereby offering a novel framework for understanding the molecular complexity of lung cancer progression.

## Data Availability

The datasets presented in this study can be found in online repositories. The names of the repository/repositories and accession number(s) can be found in the article/Supplementary Material.
